# Preoccupation as psychopathological process and symptom in adjustment disorder: A scoping review

**DOI:** 10.1002/cpp.2657

**Published:** 2021-08-28

**Authors:** David J. Eberle, Andreas Maercker

**Affiliations:** ^1^ Division of Psychopathology and Clinical Intervention, Institute of Psychology University of Zurich Zürich Switzerland

**Keywords:** adjustment disorder, preoccupation, rumination, transdiagnostic, worry

## Abstract

In the ICD‐11 diagnostic guidelines, preoccupation has been introduced as the new core symptom of adjustment disorder. Despite this essential innovation, preoccupation has so far largely been defined as rumination and worry and does not feature a distinct character as an independent symptom. In order to investigate the nature of preoccupation, various cognitive approaches are evaluated and linked to preoccupation. Furthermore, the aim of this review is to define preoccupation more precisely and to distinguish it from other symptoms in psychopathology. The evaluation of key features of cognitive dissonance theory, attention bias theory, memory theories, and other cognitive paradigms indicates that preoccupation is constituted by a complex interaction of cognitive–emotional mechanisms. In addition, this review implies that preoccupation in AjD can be defined as stressor‐related factual thinking, which is time‐consuming and often associated with negative emotions. It is assumed that rumination and dysfunctional worry serve as reactive processes to cope with preoccupation. For further distinction, this review presents similarities and differences of preoccupation and other symptoms, including negative automatic thoughts, flashbacks, and yearning. Finally, implications and suggestions for future research on preoccupation are offered. Overall, it is plausible that preoccupation is not only associated with adjustment disorder but also possesses a transdiagnostic character.

**Key Practitioner Messages**

- Preoccupation has been introduced as the new core symptom of adjustment disorder in ICD‐11
- Preoccupation can be defined as stressor‐related factual thinking, which is time‐consuming and associated with negative emotions
- Preoccupation may not be limited to adjustment disorder but possesses a transdiagnostic character

## 1 INTRODUCTION

With the introduction of the ICD‐11, the principle of defining specific core symptoms for the diagnosis of mental disorders has become the norm (WHO, 2020). Preoccupation has now been defined as *the* core symptom for adjustment disorder (AjD) (WHO, 2020). AjD has a somewhat unique position in psychopathology since, despite being one of the most frequently diagnosed mental disorders (Reed et al., 2011), it has been defined as a low‐threshold or a diagnosis of exclusion. With this new and improved disorder concept—defined by a core symptom—there is now a revived scientific and practical interest in the specific definition of preoccupation (e.g., Bachem & Casey, 2018). The present paper aims to review the state of knowledge on preoccupation and to derive a scientific working model for this symptom.

Besides preoccupation as core symptom, the current definition of AjD includes a stressor criterion (the existence of at least one stressful event in the recent past), as well as failure to adapt to the stressor. Furthermore, AjD typically resolves within 6 months, unless the stressor persists for a longer duration (WHO, 2020). Originally, it was the development of trauma and stress‐related disorders, especially posttraumatic stress disorder (PTSD), that led to the emergence of preoccupation as the novel core symptom of AjD (Maercker et al., 2007). With his concept of “stress response syndromes,” in which intrusions are a key element, Horowitz (1986) had already provided a detailed contemporary psychopathological description not only of PTSD but also of AjD and grief. Hence, preoccupation is regarded as a psychopathological “sibling symptom” of posttraumatic intrusions or re‐experiences. Following this scientific foundation, the present review examines the clinical significance of cognitive processes together with their relevance for a specific delimitation and substantive assessment of preoccupation. Beginning with a description of the lines of development towards the current concept of preoccupation in AjD, it then presents its methodological basis. This is followed by the elaboration and evaluation of relevant psychological phenomena and concludes with the presentation of a working model for preoccupation. As an example, the topic of involuntary job losses and divorces is often used, which have recently been studied more intensively (e.g., Lorenz et al., 2018; Maercker & Lorenz, 2018).

### 1.1 Stress–response syndromes

Disorders caused by a psychosocial stressor such as AjD, PTSD, and prolonged grief disorder (PGD) can, according to Horowitz's (1986) stress–response syndrome model, be localized in a stress–response continuum and follow a specific pattern of development. First, a stressful life event causes a stage of shock and realization concerning the stressor in which a strong emotional reaction arises, often followed by a denial of the new reality (Horowitz, 1997). Subsequently, when an individual recognizes the stressor with all its consequences, a period of re‐experiencing and intrusion occurs. Horowitz (1986) pointed out that ideally, after repeated occurrence of intrusions, that stressful memories become integrated into one's cognitive schemata and thus into the long‐term memory system. Due to this elaboration, intrusions fade after some time. It is assumed that this process occurs in all individuals who have experienced a stressful event, which is why Horowitz (1986) defined it as stress–response syndrome. As core symptoms, intrusions are a key element in this process. Such intrusion‐analogue symptoms can be identified in stress‐related disorders, including flashbacks in PTSD, preoccupation in AjD, and yearning/longing in PGD (in the following, yearning/longing is collectively referred to only as yearning).

### 1.2 Elaborating a new concept of the preoccupation symptom

To date, there is no generally accepted psychopathological definition of preoccupation. The Oxford English Dictionary (n.d.) offers a definition of preoccupation as a state of “thinking about something continuously and something that you think about frequently or for a long time”. This definition is close to the clinical experience of patients who cannot turn their thoughts away from a serious life event. However, the detailed characteristics of this phenomenon and how it differs from other thought processes have not yet been defined.

The ICD‐11 definition of AjD describes preoccupation as “preoccupation with the stressor or its consequences, including excessive worry, recurrent and distressing thoughts about the stressor, or constant rumination about its implications” (WHO, 2020). Thus, the phenomena of rumination and worry need to be examined more closely in terms of their similarities to and differences from preoccupation. In an attempt to summarize various definitions of rumination, Smith and Alloy (2009) concluded that rumination can be defined as a stable, negative, and broadly construed way of responding to discrepancies between current status and target status. Worry, on the other hand, has been defined as a chain of negatively affect‐laden and relatively uncontrollable thoughts and images that promote mental attempts to avoid anticipation of potential threats (Borkovec et al., 1983). Whether these symptoms really overlap with preoccupation is unclear and will also be differentiated in this review.

Furthermore, it is important to distinguish other constructs in psychology from preoccupation, such as flashbacks, yearning, and negative automatic thoughts. Repetitive and intrusive processes are recurrent phenomena in various disorders (Ehring & Watkins, 2008), which is why preoccupation is specifically discussed as a transdiagnostic symptom. Finally, all theories and ideas will be synthesized, and new features of the preoccupation symptom will be presented.

## 2 LITERATURE SELECTION PROCESS

First, we examined recent studies that investigated AjD to check for different conceptualizations and possible working models of preoccupation. For this purpose, a review of the published literature on PsycINFO and Pubmed databases was conducted, using the keywords *preoccupation* AND‐paired with *adjustment disorder*. Articles were then examined regarding the definition and conceptualization of preoccupation. This literature search revealed that since the release of ICD‐11 beta version in 2013 and the introduction of preoccupation as symptoms, only 34 studies have investigated AjD while including preoccupation. All these studies defined preoccupation according to ICD‐11 as excessive worry, recurrent and distressing thoughts about the stressor, or constant rumination about its implications, without proposing new aspects of preoccupation. Hence, there was no study that conceptualized preoccupation beyond ICD‐11 definition.

Second, a chosen range of psychological theories was examined and discussed to further clarify and explain the preoccupation symptomatology. Due to the large number of different paradigms in this field, it was necessary to limit the analysis to a few cognitive theories that had the greatest potential in explaining preoccupation after a stressful life event. Based on the consensus of involved researchers concerning the eligibility of the different paradigms, selected theories included cognitive dissonance (CD) theory (Festinger, 1962), attention bias theory (e.g., Woud et al., 2017), memory mechanisms (e.g., Mace, 2008), motivational aspects (e.g., Kelly, 1991), as well as the dynamics between cognition and emotion (e.g., Izard, 2011). Excluded paradigms were, for example, metacognitive approaches (e.g., Anyan et al., 2020), learning and development theories (e.g., Karantzas et al., 2010), assimilative and contrasting comparison effects (e.g., Smith, 2000), and cognitive salience principles (e.g., Baumeister et al., 2001). The chosen cognitive theories and their possible explanations of the preoccupation symptom are outlined in the following sections.

## 3 PREOCCUPATION AND COGNITIVE MECHANISMS

Psychopathological conditions are, to a considerable extent, rooted in cognitive mechanisms (Haywood & Raffard, 2017). More specifically, mental disorders often derive from an altered perception, transformation, elaboration, or usage of information—processes that are also key elements in cognitive approaches. Thus, as Bentall (1996) suggests that theories about cognitive mechanisms could fundamentally contribute to research in psychopathology, since they create a detailed understanding of how normal and abnormal psychological processes are organized. In addition, cognitive theories enable a broader view and new perspectives in psychopathology. Haywood and Raffard (2017) also point out that concepts from cognitive theories can directly serve to develop new classifications of psychopathological symptoms and abnormal psychological conditions in general. The following sections will provide such insights for preoccupation. We assume that preoccupation is not only a symptom but also a complex cognitive phenomenon that also constitutes a consequence and epiphenomenon of stress. However, as preoccupation has never been profoundly described before, it is hoped that cognitive theories can shed light on the diverse facets of this construct.

### 3.1 CD theory

A widely known theory which can help understand preoccupation is the CD theory (Festinger, 1962). It is assumed that cognitive consistency is a basic need of every human being and is similarly important to the need for nutrition, sleep, or safety (Festinger, 1962). Cognitive consonance is defined as a harmony or concord between cognitive elements, such as opinions, knowledge, attitudes, or basic assumptions (Festinger, 1962). This can also include cognitive representations of a reality or a behaviour, for example, if an individual pursues a professional activity. When cognitive elements are no longer in harmonical arrangement, a CD emerges. For instance, if being employed is an important value for an individual and this person is about to lose their job, then CD is expected to arise.

CD theory proclaims that CD is psychologically uncomfortable and distressing. CD often causes counteracting mental strategies with the aim of reducing it (Festinger, 1962). This can include re‐evaluation of distressing information, adding consonant cognitions, or disregarding dissonant information (Harmon‐Jones & Mills, 2019). For instance, if an individual becomes unemployed, this person could start thinking that their job was not that good anyway or that a professional re‐orientation was necessary. However, counteracting strategies tend to be less effective in cases of strong CD (Gawronski & Brannon, 2019). For example, after working in a highly satisfying job for 20 years, an individual can experience high CD after being dismissed and might not be able to simply re‐evaluate the situation. It is at least more difficult to do so compared with a case of weak CD, like creating a white lie (Cotton, 1985). If CD becomes overwhelming to a point that it cannot be erased by counteracting strategies, an individual might remain in an ongoing state of CD and distress (Festinger, 1962; Gawronski & Brannon, 2019). This state could be identified as preoccupation. The discrepancy of cognitive elements in CD is similar to AjD, in which a stressor is also in conflict with the previous life condition. In addition, an ongoing CD, caused by repeated dissonance‐generating information, is equal to the reoccurring characteristics described for preoccupation (WHO, 2020).

The idea of preoccupation as processing of dissonance is also insinuated by Horowitz (1986), who identifies certain stages of distress. The phase of intrusions is characterized by unprompted ideas as part of a processing of stressor events. After an aversive life incident, an individual is confronted with a changed and often disturbing reality. This experience is distressing, since it provides new information that is in most cases highly incongruent with pre‐existing schemata (Horowitz, 1986). This incongruence causes the mind to repeatedly process the new information until reality, and the individuals own schemata are brought into accord (Horowitz, 1986). At this stage, mental representations of a traumatic event are constantly stored in the active memory, which again creates incongruency and sets information processing in motion. It is through this process that a traumatic recollection after a stressful event can occur repeatedly (Horowitz, 1986). The stage of ongoing processing of incongruence described above could be identified as preoccupation.

Festinger (1962) as well as Stalder and Anderson (2014) point out that every person experiences CD and that only a high amount of CD is critical for mental health. If a certain threshold of CD is crucial, this might also mean that the amount of preoccupation is relevant in determining if it is pathological or not. For instance, being preoccupied over a short time due to a relatively harmless sickness, like a cold, is arguably not problematic for a person's psychosocial functioning. But being preoccupied over months because of a serious illness, like cancer, can very well be considered as pathologically relevant (Shevlin et al., 2019). It is therefore plausible that the amount of preoccupation is a key factor, just as it is the case with the amount of CD. Thus, the intensity or quantity of preoccupation needs to be considered as a defining characteristic of this symptom.

In sum, CD and preoccupation seem to be closely connected. Eventually, after a stressful life event, CD cannot be erased by re‐evaluations due to its strength and remains present. Hence, preoccupation is presumably the processing of this ongoing CD. A defining characteristic of both CD and preoccupation could be a certain duration and intensity with which these phenomena appear.

### 3.2 Attention bias theory

An attention bias is a tendency to selectively attend to personally relevant or disorder‐relevant stimuli (Beard et al., 2012). For instance, after experiencing an aversive life event, individuals tend to react differently to distressing stimuli compared with normal stimuli (Woud et al., 2017). Two important mechanisms can be identified within the attention bias theory: attention facilitation and attention interference (Pineles et al., 2007), which are also known as attention vigilance and attention maintenance (Posner & Peterson, 1990; Weierich et al., 2008). If an individual shows a vigilant and quick reaction towards a stimulus, then attention facilitation takes place. In contrast, difficulty disengaging attention from stimuli is defined as interference bias (Pineles et al., 2009).

While attention facilitation helps an individual to detect personally relevant stimuli more quickly, which is regarded as an important evolutionary mechanism (Fox et al., 2001), the role of attention interference is less clear (Pineles et al., 2009). Prominent theories suggest that an interference of attention could be the result of a cognitive network activation, which reflects a broadened processing of emotion‐based information (e.g., Foa & Kozak, 1986). This implies that attention could be interfered due to the large amount of mental activation after an individual is faced with stressful information. This could also be a mechanism in preoccupation.

An attention interference is indeed linked to a specific stress‐related psychopathology. In a study with individuals who had experienced stressful events, Wisco et al. (2013) showed that attentional interference and intrusive symptoms are closely connected to each other because both include a tendency to cognitively remain on a stressor (Wisco et al., 2013). Furthermore, clinical studies show that the inability to withdraw attention from intrusions leads to the development of a strategy of thought control and suppression, as an attempt to cope with unwanted thought content (Nixon et al., 2009; Wisco et al., 2013). For instance, after being informed about suffering from a severe disease, an individual might suppress thoughts about the illness or heavily start controlling thought processes due to the emotional distress of such cognitions. This could be regarded as a reaction to intrusion and thus a reaction to preoccupation. Therefore, it can be assumed that intrusive symptoms, such as preoccupation, are significantly influenced or predominated by avoidance mechanisms (see also Wisco et al., 2013).

In sum, preoccupation may be regarded as an interfered attention bias. Small cues like thinking about the stressor can cause an immersive and ongoing activation of cognitive networks, leading to a cognitive remaining on thought content related to the stressor. Such a network activation possesses an intrusive character and is emotionally aversive due to the remaining cognitive confrontation with the stressor.

### 3.3 Everyday memory processes

The human memory system contains a vast number of interlinked memories (Anderson & Bower, 2014). Thinking back on a specific life event can elicit numerous associated memories due to an interlinked retrieval. Therefore, after a stressor is cognitively activated, it can cause a chain reaction in the memory retrieving process. For instance, the retrieval of a divorce might not only trigger the moment when the breakup happened but it may also elicit memories of the further divorce procedure or memories of the first days without the partner and so on. This way, memories are triggered through other memories, leading to a vicious cycle. Such a mechanism was also shown in autobiographical memory research: Retrieval processes often work in conceptual lines (Mace, 2014). This means that memories have a natural tendency to trigger other memories of the same conceptual or experiential type, such as memories from the same stressor. A memory chain reaction eventually works in parallel with other mental activation phenomena, such as an emotional network activation (e.g., Foa & Kozak, 1986). However, in the case of stress‐related conditions, a specific memory activation is especially relevant for pathological explanations (Brewin, 2011).

When a behaviour is repeatedly executed, more and more memory representations of this behaviour develop, as a person continues making and elaborating experiences (Crowder, 2014). For example, if an individual works in the same job for 20 years, far more memory representations exist concerning the past profession compared with a person who only worked for 1 month in the same job. After an aversive event, a past behaviour is often not executed anymore (e.g., not working anymore in the past job). However, the memory network of these past experiences still exists as memory representations. What then might happen is that this person is frequently reminded of the past job because of all the memory conjunctions. This can mean that the morning coffee, sitting at a desk, or the lunch break reminds an individual of activities of their past job, since this is how these reminiscences are associated in the memory system. An entire pattern of automatic and interlinked thoughts can still be in place, even though life may have changed fundamentally. For individuals with AjD, this likely becomes constantly distressing since they are frequently reminded of their past lives. The very process of constant remembering is arguably an amplifying mechanism for preoccupation.

In short, willingly, or unwillingly retrieved memories of the aversive event can cause a chain reaction of stress‐related recollections. Furthermore, an extended memory network from the time before a stressful event, which is still active and connected to present cues, is probably a hotbed in which preoccupation can unfold a large impact on an individual.

### 3.4 Additional motivational aspects

Many individuals are shattered and disoriented after stressful experiences (Janoff‐Bulman, 1992). However, reprocessing information from such experiences is an important step to recovery (Creamer et al., 1992). Due to the significant implications of aversive events for the course of their own life, there is a motivating component for individuals with AjD to think about the stressor repeatedly. Acquiring a deep understanding of the contexts, antecedents, and reasons for distressing experiences as well as drawing conclusions from them could be considered as a natural urge after distressing experiences. For example, a person who unwillingly lost their job may be so deeply overwhelmed by the new situation that they have a strong need to understand what happened and what implication this event has, for example, realizing that everyday habits or the financial situation have fundamentally changed. Immediately after an aversive life event, when the stressor is very recent and implications of the event are not yet clear, individuals may show a particular motivation to clarify and understand the new circumstances. Related theories of this urge to reprocess information are personal and social constructivist approaches (e.g., Gergen, 2011; Kelly, 1991). Experiences are subject to personal and automatic construction processes, for example, to make sense of the world (Kelly, 1991; Walker & Winter, 2007). Events that are difficult to make sense of, such as an unexpected and distressing divorce, might also lead to persistent preoccupation.

Reflecting information and anticipating further consequences can be an underlying feature of preoccupation. However, a reflection process is also considered as part of other cognitive processes, such as rumination. Eventually, rumination steps into this process as negative way of responding to implications from an event (Smith & Alloy, 2009). For example, reflecting the loss of a job and its implications could be labelled as preoccupation. As a reaction to this process, a negative evaluation of the situation or of life in general could be caused, which represents rumination. Reflecting a job loss can also leave space for worry. If an individual realizes that the financial situation can be very tough after the job loss, worry could become very dominant. Importantly, this would be an additional step in the process. The reflection itself is the primary action, eventually followed by reactions to this process. Not all individuals show an ongoing tendency to worry or ruminate; a person can also just perceive the job loss and become optimistic about the future, even though it is a distressing and painful situation. Hence, the motivation and urge to process information could be an important factor in the induction of preoccupation, which also leaves space for other symptoms to interfere, such as rumination and worry.

### 3.5 Cognitive–emotional dynamics

Different modalities of human functioning, such as emotions, cognitions, behaviour, and physiology, are highly interconnected and influence each other (Izard, 2011; Izard et al., 1984). Cognitions can alter physiological and emotional states even through simple cognitive exercises (Beck, 1995; Beck & Tompkins, 2007; Wilson et al., 2004). Emotions also have an impact on an individual on several levels (Lench et al., 2011; Stemmler, 2004). For example, a person who feels angry after the loss of their job may experience changes on a behavioural level, resulting in restlessness, as well as changed physiological components, like an altered heartbeat and trembling. Emotions can also cause a change in cognitions (Borkovec, 1985; Izard, 2011; Okon‐Singer et al., 2015). For instance, after the loss of a job and the emotional arousal due to this event, job‐related cognitions might become very prominent merely due to this emotional arousal, which is cognitively perceived and processed.

Studies have shown that after a stressful life event, many individuals with AjD perceive intensive and distressing emotions (e.g., Anaf et al., 2013; Blau, 2006; Brewington et al., 2004). As a triggered cognitive component of this emotional distress, preoccupation may form. The emotional pain might be so dominant that it could be difficult not to think about it. This phenomenon can be observed in people with chronic pain: A study with patients suffering from a chronic pain disorder showed that the feeling of pain also triggers attention and thoughts related to pain (Crombez et al., 2013). In other words, emotions trigger a cognitive focus on the current state. Thinking, on the other hand, could bring the aversive event to the attention again, which could elicit negative emotions once more. As a result, both processes, emotions facilitating cognitions and vice versa, may result in a cognitive–emotional dynamic, which finally forms preoccupation as persisting, ongoing and distressing symptom, as it is defined in the ICD‐11.

## 4 INTERMEDIATE SUMMARY

Preoccupation has been depicted from various perspectives and with different cognitive approaches. In CD theory, the discrepancy between cognitive contents is realized and reflected. For example, the unwanted loss of a job may be incompatible with the values of pursuing a job. This dissonance is perceived with fact‐based thoughts such as “I am now unemployed” or “I now need to find a new job to earn money”. This could be called factual thinking. Such a persistent cognitive processing is similar to an attention bias, which is a systematic tendency to focus on disorder‐relevant information. In case of AjD, it is therefore plausible that preoccupation is specifically focused on stressor‐related information. Repeated thinking about the stressful life event might also cause further memory activation. For instance, the thought “I have lost my job” can produce related memories such as “I have always enjoyed my work” or “My partner also unintentionally lost his job 5 years ago”. Furthermore, an unresolved stressor additionally motivates thinking about the aversive life event to understand and draw conclusions from what has happened. For example, a job loss may trigger thoughts such as “The economic situation is responsible for the loss of the job.” These cognitive mechanisms induce a persistent preoccupation with the stressful life event. Preoccupation should therefore be defined as time‐consuming phenomenon. Furthermore, it has been proposed that stressor‐related thoughts trigger negative emotions. These aversive emotions can in turn evoke stressor‐related cognitions. For example, the pain after a divorce could be processed factually with thoughts like “I will never feel the closeness of my partner again!”. Therefore, it is reasonable to assume that preoccupation is often associated with negative emotions.

Thus, preoccupation in AjD can be defined as stressor‐related factual thinking, which is time‐consuming and often associated with negative emotions. In this context, factual thinking is best described as fact‐based thoughts about the stressor, which are neutral in content. Furthermore, factual does not mean that thoughts always need to be consistent with reality: Hypothetical scenarios, such as “If I were still together with my partner, I would go to work with her now” might also be considered as “factual.” This preliminary definition of preoccupation illustrates that preoccupation possesses a unique character, which is further differentiated in the next sections.

## 5 DIFFERENTIATION OF PREOCCUPATION TO RELATED PHENOMENA

In the current ICD‐11 definition, preoccupation is strongly associated with rumination and worry. As an intrusive symptom, preoccupation is also closely related to other core symptoms of stress‐related disorders, such as yearning or flashbacks. The following sections shed light on the relationship of preoccupation to cognate psychological constructs.

### 5.1 Repetitive thoughts

Repetitive thoughts (RT) are defined as the process of thinking attentively, repetitively, or frequently about oneself and one's world (Segerstrom et al., 2003). RT represent a general process of thinking and no specific cognitive symptom. Thus, RT can include many ways of thinking or various symptoms, including studying, rumination, worry, and planning. Valence is not determinative; hence, RT can have positive, neutral, or negative valence (Smith & Alloy, 2009). It seems obvious that preoccupation, as time‐consuming, factual thinking, also falls under the category of RT. The conceptual difference with RT may be that RT is an umbrella category of cognitive phenomena, with preoccupation representing one of these phenomena.

### 5.2 Rumination

In clinical psychology, rumination is a common symptom associated mainly with depression but also with anxiety disorders (Smith & Alloy, 2009). There are a variety of theories and approaches to defining rumination (for an overview, see Smith & Alloy, 2009). In summary, rumination can be defined as a stable, negative, broadly constructed way of responding to discrepancies between the current status and the target status (Smith & Alloy, 2009). To date, rumination has been a defining component of the preoccupation symptom in AjD (WHO, 2020), and indeed, there are many similarities between these two constructs. Rumination can be understood as a type of RT (Smith & Alloy, 2009), which is also the case for preoccupation. Moreover, both latter constructs are likely to be associated with negative emotions and with a discrepancy between the current state and the target state. Despite this, on closer examination, there are considerable differences between the two concepts.

An example of a ruminative thought is the statement “Why am I such a failure?!” (Hirsch & Mathews, 2012). A negative appraisal or response to differences between current status and target status is a decisive criterion of rumination (Papageorgiou & Wells, 2001; Smith & Alloy, 2009). This negative appraisal or response is shown as negative thoughts (Papageorgiou & Wells, 2001). At this point, a first difference to preoccupation can be identified: While rumination contains negative thoughts like “This disease and everything related to it is terrible,” preoccupation is defined by factual thoughts like “I suffer from a disease.”

Rumination is often defined as avoidance coping strategy (Ehlers & Steil, 1995; Foa & Kozak, 1986; Matheson & Anisman, 2003; Matthews & Wells, 2004; Michael et al., 2007; Segerstrom et al., 2003; Smith & Alloy, 2009). After a job loss and to avoid thinking about never working in the former job again, an individual might start to ruminate and to think about how bad the world is in general. This way, a refocusing of attention takes place, which is emotionally less painful than thinking about the stressor. Research shows that the avoidance mechanism in rumination can be very subtle. For example, Ehlers and Steil (1995) found that rumination works avoidantly just by focusing on the verbal channel or by the more abstract level of thinking that characterizes this symptom. It is unplausible that this avoidance mechanism also exists in preoccupation. The pure processing of the stressor with factual thoughts could be described as a main feature of preoccupation. As another example, studies with PTSD patients show that individuals use rumination to avoid intrusions of the trauma (e.g., Michael et al., 2007). Analogously, it is very plausible that this rumination avoidance mechanism also occurs when individuals are constantly preoccupied with intrusive thoughts of their aversive life event. Therefore, the avoidance function presumably constitutes another difference between preoccupation and rumination.

Furthermore, preoccupation can probably not be considered as way of responding to discrepancy between current and target status, as it is the case for rumination (Smith & Alloy, 2009). Preoccupation appears to be the discrepancy‐related (or dissonance‐related) thinking to which rumination negatively responds. In other words, preoccupation could be a stressor‐related thinking and if this process is too distressing, then negative response and avoidance strategies may take place, such as rumination. After an aversive event, individuals may be more concerned with cognitions like “Why am I such a failure?!” rather than having to deal with the actual stressor. In the discussion of preoccupation, it is essential to consider that a stressor does not always have to be followed by a negative response to the stressor. An individual can also process a stressor factually as preoccupation rather than falling into negative ruminative thinking. Similarly, other reactions to stress are conceivable, such as optimism.

In short, the most important distinguishing features between preoccupation and rumination are arguably the different content of the thoughts (factual for preoccupation and negative for rumination) and the dynamics of the two symptoms (rumination as avoidance of preoccupation). Therefore, it could be concluded that preoccupation and rumination are two different symptoms. Further implications to this conclusion follow below.

### 5.3 Negative automatic thoughts

A very subtle cognitive phenomenon are negative automatic thoughts (NATs), which are defined as immediate and negative evaluations people make about their situation, themselves, or their future (Flouri & Panourgia, 2014). An example of a NAT is “I am stupid!”. NATs are a common phenomenon in all individuals but frequently associated with depression and rumination (Kumari & Blackburn, 1992). The main difference between NATs and rumination lies in the quantitative extent of these constructs. Whereas NATs are short‐lasting, negative evaluations, rumination is characterized by an entire pattern or chain of ongoing, negative evaluations, which distinguishes these two symptoms (Papageorgiou & Wells, 2001).

Similarly to rumination, preoccupation could be different from NATs concerning the content of thoughts. While NATs are defined by negative cognitions, preoccupation is probably characterized by factual thoughts. In addition, preoccupation is likely to be distinguished from NATs by the quantitative extent. It is plausible that NATs and preoccupation are often mixed. For example, after a divorce, a chain of thoughts can occur such as “Since the divorce from my partner, I live alone in my flat. This is just terrible! Exactly two months ago, the divorce took place.” What can be perceived as a negative chain of thoughts is more likely to be a processing of facts in which a quick, negative appraisal is incorporated. It is important to distinguish the constructs contained therein, as they have a different, psychopathological basis. The aforementioned chain of thoughts could mainly contain preoccupation but be complemented by NATs and therefore be considered as rumination and later diagnosed as depression. In order to avoid such a misconception, which could have serious consequences such as a changed diagnosis and therapy, close examination and differentiation of these constructs are strongly needed.

### 5.4 Worry

Worry is a construct that refers to different conceptualizations. One widespread definition describes worry as a chain of negatively affect‐laden and relatively uncontrollable thoughts and images that promote mental attempts to avoid anticipation of potential threats (Borkovec et al., 1983). Overall, an apprehensive expectation can be regarded as a central element of worry (APA, 2013). Worry typically occurs in Generalized Anxiety Disorder (GAD) and also in many other anxiety‐related conditions (Hirsch & Mathews, 2012). An exemplarily thought of worry is “What if something terrible will happen?!”. Thus, preoccupation differs here in the content of thoughts, which is factual compared with worry thoughts in the form of anxiety‐based expectations. An example of differentiation is the reaction to a job loss: Worrying individuals may have thoughts like “What if I run out of money and lose my flat?”, while preoccupied thoughts are arguably factual, like “I will have less money available next month”. Furthermore, an important characteristic of worry is a future‐orientation (Papageorgiou & Wells, 1999). Preoccupation can probably be future‐oriented and also focused on the present or the past. It is almost obvious that in AjD and a state of preoccupation, a person also thinks of a situation in the past, such as the aversive event that caused the AjD. The temporal orientation is thus a fundamental distinguishing feature between preoccupation and worry.

Worry thoughts can be helpful or dysfunctional. Worry can protect people from threats and be adaptive once these cognitions are solution oriented (Querstret & Cropley, 2013). When worry becomes uncontrollable or persists despite failed attempts at resolution, it can be considered as pathological or dysfunctional (Hirsch & Mathews, 2012). There is a consensus that persistent and goalless worry can be regarded as avoidance of aversive psychological states (Borkovec et al., 1998; Michael et al., 2007; Smith & Alloy, 2009; Wisco et al., 2013). For example, if a person is having relationship problems, that person might start worrying about financial matters instead, in order not to face stressful facts or problems in the relationship. Thus, worry could function as a coping strategy to avoid a distressing preoccupation, as it is arguably the case with rumination.

In summary, it is most likely that preoccupation (factual thinking) and worry (anxiety‐based thinking with exclusive orientation into the future) are distinct symptoms. It is plausible that dysfunctional worry represents an avoidance strategy as a reaction to preoccupation.

### 5.5 Everyday thinking

As factual thinking, preoccupation also raises the question of how to distinguish it from neutral, everyday thinking. Such thinking is often researched in nonclinical populations (e.g., Baumeister et al., 2020; Behar et al., 2012). In terms of content, everyday neutral thinking is probably very similar to factual thinking in preoccupation. For example, a person who has been diagnosed with a terminal illness may think “I am ill and I will die from my disease.” This does not necessarily trigger a significant emotional response in all individuals. Maybe a person has lived his or her life satisfactorily and is relatively indifferent to such a thought. However, concerning individuals with AjD, such a thought may trigger intense, negative emotions. Individuals without AjD may also be affected by such a thought. However, the difference to individuals with AjD might be that the latter show a significantly stronger reaction to these cognitions. A certain threshold of an emotional reaction could thus be identified as a distinguishing criterion between neutral thinking in individuals with and without AjD.

In addition, neutral thinking in individuals with and without AjD possibly differs in the focus of thinking. While individuals without AjD eventually do not show a specific focus of thoughts, cognitions of individuals with AjD could very often be focused on a stressor. In other words, it could also be the case that individuals with AjD think about a stress‐related topic in a factual way for a longer period of time than individuals without AjD. For example, individuals without illness could think about the own health status but arguably not as extensively as patients with a terminal illness and AjD. This also means that factual thinking about the stressor in individuals with AjD must be defined as very time‐consuming.

### 5.6 Yearning

Yearning is, along with preoccupation, the core symptom of PGD and therefore of particular significance for the definition of preoccupation. Yearning can be defined as unsatisfied, intense, as well as future‐oriented appetitive desire towards a lost person (Eisma et al., 2020). Importantly, yearning is considered to be a cognitive–emotional process (Boddez, 2018; Eisma et al., 2020; Kaplan et al., 2018; O'Connor & Sussman, 2014; Robinaugh et al., 2016). The cognitive component of this process includes repetitive thinking about the loss (Eisma et al., 2020) which is frequently directed at the deceased person, the death of this person and its circumstances, the self, or relevant others (Neimeyer et al., 2019). The strong link between yearning and preoccupation is also indicated in the yearning in situations of loss scale (YSL), where the cognitive item of yearning can be classified as preoccupation (Robinaugh et al., 2016).

There are many more similarities between preoccupation and yearning. Both symptoms can be considered as emotionally aversive. Furthermore, many studies imply that rumination is an avoidance strategy for coping with grief and yearning (Boelen et al., 2006; Eisma et al., 2015; Morina, 2011; Stroebe et al., 2007), which is arguably also the case for preoccupation. Regarding AjD as a whole, emotional responses to aversive life events are common phenomena. For instance, after a job loss, emotions such as despair, shame, or anger often emerge (Anaf et al., 2013; Brewington et al., 2004). As another example, Linden et al. (2012) showed that being diagnosed with cancer can elicit various anxiety responses, depending on the specific type of cancer. Such responses to stressors can also take the form of grieving or yearning: Studies have shown that after a job loss, many individuals go through some sort of bereavement process (Anaf et al., 2013; Blau, 2006; Brewington et al., 2004). Yearning has also been described in the context of divorces and family breakups (Kruk, 1992, 1994), which are typical cases of AjD. Among the various reactions to different stressors described above, yearning could represent one specific subform of emotional reactions. Finally, as cognitive component of such states, preoccupation might be identified as the overlapping feature of such emotional conditions.

Other differences between yearning and preoccupation might include that yearning in PGD is specifically focused on a loved but deceased individual. Because of the inability to ever be in contact with the deceased person again, yearning in PGD perhaps includes a unique feeling of loss, which differentiates it from other stress‐related symptoms. In addition, yearning could be defined as emotion‐based, whereas preoccupation often appears more cognition‐based. It can be concluded that even though yearning represents a separate symptom from preoccupation, it is plausible to assume that yearning and preoccupation share a psychopathological base.

### 5.7 Flashbacks

Flashbacks are another intrusive symptom occurring in disorders specifically associated with stress, which makes it an important symptom which preoccupation needs to be differentiated from. Flashbacks can be defined as “the involuntary intrusion of vivid and detailed images in which the traumatic scenes are reexperienced as though they were occurring in the present” (Brewin, 2014, pp. 69). Even though flashbacks are mainly associated with PTSD and complex PTSD (in the following, CPTSD is always included when referring to PTSD), they can also appear in few other disorders, such as dissociative amnesia (Bryant et al., 2011). Flashbacks are assumed to occur because a traumatic situation has been increasingly processed by perceptual memory structures and less by episodic memory mechanisms (Brewin, 2014). This results in a specific type of trauma memory, which is responsible for the emergence of flashbacks (Brewin, 2014).

Flashbacks and preoccupation can both be regarded as intrusive symptoms. However, flashbacks feature a unique character compared with other forms of intrusions. For example, they show a particular “nowness” and are considered especially vivid, for example, by featuring particularly high perceptual details (Brewin, 2014). Empirical studies indicate that flashbacks are specifically associated to PTSD (Bryant et al., 2011) which already demonstrates a difference to intrusive symptoms in AjD, namely, preoccupation. In the form of intrusive imagery, flashbacks also differ from intrusive thoughts in their character of modality. In addition, compared with an ongoing preoccupation, flashbacks are relatively brief (Ehlers & Steil, 1995). Even though there is still an ongoing debate about how intrusive symptoms should be differentiated and what basis they have in the memory system, there is repeated evidence that intrusive symptoms of individuals with PTSD differ substantially from intrusive symptoms in people who experienced an aversive life event but do not have PTSD (Berntsen et al., 2003; Birrer et al., 2007; Reynolds & Brewin, 1998). This is yet another indicator that when PTSD develops, different intrusive mechanisms are present compared with individuals who experienced an aversive life event, but do not have PTSD, like individuals with AjD. The trauma memory with its specific alterations in episodic and perceptual memory could be the source of this difference because there seems to be a specific relation between these memory mechanisms, flashbacks, and PTSD (for an overview, see Brewin, 2014).

In this review, preoccupation has been explained mainly by using cognitive–emotional paradigms, without including a trauma‐specific memory as cause for preoccupation. A trauma‐specific memory is assumed to be the result of extreme stress during a traumatic event, which has effects on the perceptual and episodic memory (Brewin, 2014; Metcalfe & Jacobs, 1998). In cases of AjD, however, stress might not be strong enough to cause such alterations in the memory systems. Thus, it is questionable if preoccupation is caused by an altered perceptual and episodic memory. Subclinical examples additionally support a separation between preoccupation and a trauma‐specific memory. If an altered episodic and perceptual memory is responsible for preoccupation, then this trauma‐specific memory would need to be in place for every case of mild preoccupation. For example, in healthy individuals, slowly arising, financial difficulties could cause mild stress and lead to a moderate preoccupation about their financial situation. It is very unlikely that such a mild type of preoccupation is caused by a trauma‐specific memory. A fluctuating preoccupation can be far better explained by the dynamics of cognitive–emotional processes.

In sum, there is evidence that intrusive symptoms in PTSD possess a unique character. For instance, flashbacks feature a particular nowness, caused by memory mechanisms that are specifically related to extremely stressful and traumatic events. As conclusion, an altered episodic and perceptual memory is eventually the major distinguishing feature between flashbacks and preoccupation.

## 6 A NEW DEFINITION OF PREOCCUPATION

As a conclusion of this review, we propose four defining characteristics for preoccupation in AjD:

- Preoccupation contains factual (neutral) thoughts
- Thoughts in preoccupation are stressor‐related
- Preoccupation is time‐consuming
- Preoccupation is often associated with negative emotions

Several other features are additionally decisive. Theoretical considerations that are outlined in the differentiation to other symptoms indicate that the temporal orientation of preoccupation can be in the past, present, or future. Moreover, it is likely that preoccupation is triggered not only by one of the described cognitive processes but also by the interaction of several of these mechanisms and represents a complex, cognitive–emotional construct (e.g., Izard, 2011). In addition, symptom differentiation showed that preoccupation is closely related but pathologically different from rumination and worry in many aspects, for example, in the content of thoughts. As frequently shown in research, rumination and dysfunctional worry serve an avoidance purpose and, in the case of AjD, are a reaction to preoccupation as avoidance or coping strategy. Depending on the individual characteristics of a person, preoccupation, worry, and rumination are probably in continuous interaction and take on a complex, cognition‐like appearance, which is strongly fluctuating. It is also quite conceivable that other symptoms and cognitive states (e.g., thought suppression or optimism) are mixed into this cognitive manifestation and serve a similar purpose (e.g., avoidance or coping) as rumination and worry. A frequent interaction and co‐appearance of symptoms is no rare phenomenon. In fact, this hybrid appearance of symptoms is rather common; for example, rumination and worry are often described as appearing in a mixed form, for example, in depression (Smith & Alloy, 2009).

## 7 DISCUSSION

In this paper, a review of cognitive mechanisms and symptoms has been presented, leading to a model of preoccupation as an independent symptom in clinical psychology. With the inclusion of specific symptom criteria, preoccupation has obtained a novel character and differs from symptoms that were previously considered to be inherent constructs. One implication is that a more thorough definition of preoccupation needs to be reformulated beyond its brief definition in ICD‐11. In particular, the relationship to rumination and worry needs to be reconsidered.

Even though our review presents an outline of the nature of preoccupation and offers specific features that define this symptom, many aspects remain still somewhat blurry and ambiguous. In particular, the influence of the presented cognitive mechanisms for the development and maintenance of preoccupation, the interaction of preoccupation with many other symptoms, as well as conclusions of the definition presented need to be examined further. The following sections will systematically address many open points.

### 7.1 Theoretical conclusions

Many features of preoccupation can still only be explained rudimentarily. For example, it is not clear what function or benefit preoccupation could have or whether there is a function at all. In clinical psychology, a specific function can be identified for many symptoms. For instance, worry can ensure prevention of potential threats. Concerning stress‐related disorders, the function of intrusion symptoms is less clear. It is assumed that in PTSD, the integration of the traumatic event into one's biography and memory structures is a key element in the recovery process (Powers et al., 2010). Similarly, in PGD, accepting and integrating the death of a loved person into one's biography is an important part of grief work (Boelen et al., 2006). Consequently, in AjD, the integration of an aversive event into one's autobiographical knowledge base could also be an important part of recovery from aversive events, similarly to PTSD and PGD. Intrusion symptoms in general and preoccupation with its repetitive processing in particular could serve exactly this function. The repeated processing and integration of an aversive event into one's biography could also explain why AjD usually resolves over time.

This review suggests that after an aversive life event, many cognitive and emotional mechanisms are set into motion, resulting in a distressing preoccupation. Preoccupation could therefore be regarded as a relatively direct result of an aversive life event. This is an important implication for the overall conception of AjD, which is also characterized by failure to adapt. Levin et al. (in press) examined the correlates of symptoms in AjD and concluded that preoccupation can be considered the central symptom of AjD. This finding underscores the significance of preoccupation in AjD, which indicates that failure to adapt could emerge as a consequence of preoccupation. For example, after a divorce, a person might think about the stressor to such an extent that everyday work becomes more and more affected until the entire professional life is impaired. From an outside perspective, this could be perceived as a failure to adapt even though preoccupation might be the main cause for these difficulties to adapt. In addition, failure to adapt is part of other stress‐related disorders as well. For instance, individuals with PTSD are characterized by an impairment in various areas of life (Jellestad et al., 2021). Such findings indicate the possibility that intrusion symptoms could occur as a direct result of stressful life events and represent core symptoms of stress‐related disorders, whereas other impairments, such as failure to adapt, are a secondary consequence of stressful experiences.

As intrusive symptom, preoccupation should also be defined as part of a larger syndrome group: The concept of preoccupation as cognitive process and the existing model of a trauma‐specific memory for the emergence of flashbacks argue for a two‐dimensional model of intrusive processes. On the one hand, there is a dynamic, cognitive–emotional basis for preoccupation as intrusive process; on the other hand, there is an alteration in episodic and perceptual memory, leading to a trauma‐specific memory system as basis for flashbacks as an intrusive process. This way, a rough subdivision of intrusive processes could be made. Different disorders might also be associated with these dimensions. PTSD is arguably more related to flashbacks, whereas preoccupation is more associated with AjD. However, for some cases of stress‐related conditions, it is also conceivable that a combination of cognitive–emotional and trauma memory mechanisms occurs. For instance, experiencing a serious accident with injuries and other effects on health could cause flashbacks from the accident as well as preoccupation due to the changed health circumstances or due to other implications of the event. Such a two‐dimensional model could offer an orienting taxonomy of intrusive symptoms, allowing clinicians to differentiate and classify such symptoms more easily. Since these intrusive symptoms probably have a different, psychological basis, their indication for treatment might become considerably simplified by such a model.

### 7.2 Transdiagnostic considerations

The conceptualization of preoccupation and differentiation from other symptoms has a transdiagnostic significance. Preoccupation could be regarded as a general stress symptom that occurs in other mental disorders and also in nonpathological conditions. As described above, preoccupation probably plays an important role in PGD and PTSD. With preoccupation as a core symptom, PGD would specifically be affected by the redefinition of preoccupation proposed here. A transdiagnostic significance may also be present for disorders that are not specifically associated with a negative life event, as repetitive cognitions are a fundamental phenomenon of many disorders (Ehring & Watkins, 2008). It is therefore plausible that preoccupation additionally occurs in depression, anxiety disorders, obsessive–compulsive disorder, or eating disorders. For instance, individuals with anorexia nervosa are defined by preoccupation with food (WHO, 2020) which might feature the same characteristics as preoccupation in AjD. In general, it is possible that preoccupation is the starting point and underlying construct of other cognitive symptoms, as it has been described above for rumination.

Various cognitive mechanisms have been presented in this review. It can be assumed that only a certain quantity or intensity of the described cognitive mechanisms causes a significant psychopathology and not the mechanisms per se. For example, CD is an everyday phenomenon in healthy people and only the quantity or intensity leads to pathological consequences (Festinger, 1957). Consequently, it is likely that preoccupation emerges broadly in a subclinical context, for example, after a failed exam. Furthermore, it seems reasonable that preoccupation develops with a different valence: The described cognitive mechanisms (e.g., mental activation due to an interlinked memory retrieval) could, for example, originate in a state of love. Positive emotions and continuous interconnection of thoughts about a specific topic, for example, about a loved person, are eventually a form of “positive preoccupation.” In addition, a type of “neutral preoccupation” is conceivable. This could occur when a person is preoccupied with a specific activity, such as a professional task, which takes up a lot of time and resources. Valence and subclinical considerations both illustrate that the transdiagnostic potential of preoccupation could reach far beyond clinical dimensions.

### 7.3 Strengths and limitations

By presenting many different approaches and angles to look at preoccupation, our review fosters a more elaborated understanding and discussion of this multifaceted symptom. Relating preoccupation to cognitive theories and other symptoms reveals not only numerous interlinks of relevant processes in AjD but also for cognitive and clinical psychology as a whole. The presentation of this broad range of ideas, however, also entails some limitations.

All studies and ideas that have been presented are based on a subjective evaluation and literature selection. There are various other theories that could explain the nature of preoccupation, such as learning and development approaches (e.g., Karantzas et al., 2010). Regarding the symptom differentiation, we only focused on common and major symptom definitions. Many more concepts would have been interesting to implement into the discussion, for example, the stage model of worry by Tallis and Eysenck (1994). The relevance of all illustrated ideas needs to be evaluated by each and every reader.

### 7.4 Implications for research and psychotherapy

Many aspects of our theoretical considerations can be broken down to implications in research and clinical practice. Regarding future research, it seems essential to reconsider the conceptualization of current symptoms in clinical psychology. Due to overlapping psychopathological concepts, researchers should reflect thoroughly how they operationalize and measure symptoms related to repetitive thinking. For an advanced understanding of preoccupation, it could be particularly fruitful to investigate specific features of this symptom. For instance, the nature of factual thinking or specific cognitive mechanisms as triggering factors for preoccupation should be studied in detail. This would help not only to understand preoccupation more profoundly but also to better operationalize it for future studies. More empirical contributions, such as new assessment tools, as well as theoretical articles and discussions are all vital to shed more light on this novel and unexplored symptom. Furthermore, clinical studies on preoccupation should be conducted not only with individuals with AjD but also with people suffering from other disorders (e.g., depression, GAD, or eating disorders), to clarify the transdiagnostic potential of preoccupation. Finally, it is possible that preoccupation represents a generic risk factor for the development of psychopathology. Investigating the nature of this symptom could therefore help understand psychopathological mechanisms more generally.

This review also entails practical implications. It has been repeatedly shown that preoccupation represents a natural process after experiencing an aversive life event. Accordingly, patients who suffer from a distressing experience should be reconfirmed that repetitive and distressing thoughts are a normal way of reprocessing such events. Although the function of preoccupation remains debatable, it is likely that this symptom serves to integrate an aversive experience into one's own biography. Cognitive theories further imply that a deeper understanding of aversive experiences is important to overcome persistent preoccupation. In conclusion, clients should be supported to reprocess and understand their experiences in order to integrate them into the own biography and to derive meaning from them (see also Neimeyer et al., 2019). Moreover, there is repeated indication that preoccupation fades away naturally after some time. However, theoretical considerations as well as empirical studies also highlight that a dysfunctional handling of difficult thoughts has a negative influence on the recovery process. Therefore, it seems important that clients do not engage in ruminative thinking processes or avoidance in general. Instead, an accepting attitude for distressing reminders as well as an adaptive thinking style should be promoted.

### 7.5 Final conclusions

In sum, this review shows that preoccupation is embedded in a dynamic system of different, psychological phenomena. The illustration of cognitive theories as well as differentiation from other symptoms has produced unique characteristics of preoccupation that enhance understanding of this symptom. Many of the presented ideas, however, remain only assumptions, which need to be tested empirically. Nevertheless, a new conception of preoccupation seems necessary. If preoccupation is simply regarded as rumination or worry, a stress‐related condition may be classified as depression or GAD, rather than a stress‐related disorder, which might entail a different, and perhaps problematic, therapeutic procedure. Ultimately, the outlined considerations could help to provide important discriminatory guidance for preoccupation, which can cause significant improvements for individuals who experienced a stressful life event.

## CONFLICT OF INTEREST

No conflict of interest was reported by the authors.

## Data Availability

No empirical study was conducted for this review.
